# Evaluation of a collaborative care model for integrated primary care of common mental disorders comorbid with chronic conditions in South Africa

**DOI:** 10.1186/s12888-019-2081-z

**Published:** 2019-04-03

**Authors:** Inge Petersen, Arvin Bhana, Lara R. Fairall, One Selohilwe, Tasneem Kathree, Emily C. Baron, Sujit D. Rathod, Crick Lund

**Affiliations:** 1 Centre for Rural Health, Howard College, University of KwaZulu-Natal, Durban, South Africa; 2 Centre for Rural Health, Howard College, University of KwaZulu-Natal; Health Systems Research Unit, South African Medical Research Council, Durban, South Africa; 3 Knowledge Translation Unit, University of Cape Town, Cape Town, South Africa; 4 Alan J Flisher Centre for Public Mental Health, Department of Psychiatry and Mental Health, University of Cape Town, Cape Town, South Africa; 5 London School of Hygiene and Tropical Medicine, London, United Kingdom; 6 Centre for Global Mental Health, Institute of Psychiatry, Psychology and Neuroscience, King’s College London, London, United Kingdom

**Keywords:** Common mental disorders, Task sharing, Collaborative care, Chronic care, Lay counsellors, South Africa

## Abstract

**Background:**

The rise in multimorbid chronic conditions in South Africa, large treatment gap for common mental disorders (CMDs) and shortage of mental health specialists demands a task sharing approach to chronic disease management that includes treatment for co-existing CMDs to improve health outcomes. The aim of this study was thus to evaluate a task shared integrated collaborative care package of care for chronic patients with co-existing depressive and alcohol use disorder (AUD) symptoms.

**Methods:**

The complex intervention strengthened capacity of primary care nurse practitioners to identify, diagnose and review symptoms of CMDs among chronic care patients; and implemented a stepped up referral system, that included clinic-based psychosocial lay counsellors, doctors and mental health specialists. Under real world conditions, in four PHC facilities, a repeat cross-sectional Facility Detection Survey (FDS) assessed changes in capacity of nurses to correctly detect CMDs in 1310 patients before implementation and 1246 patients following implementation of the intervention at 12 months; and a non-randomly assigned comparison group cohort study comprising 373 screen positive patients with depressive symptoms using the Patient Health Questionnaire-9 (PHQ9) at baseline, evaluated responses of patients correctly identified and referred for treatment (intervention arm) or not identified and referred (control arm) at three and 12 months.

**Results:**

The FDS showed a significant increase in the identification of depression and AUD from pre-implementation to 12-month post-implementation. Depression: (5.8 to 16.4%) 95% CI [2.9, 19.1]); AUD: (0 to 13.8%) 95% CI [0.6–24.9]. In the comparison group cohort study, patients with depressive symptoms having more than a 50% reduction in PHQ-9 scores were greater in the treatment group (*n* = 69, 55.2%) compared to the comparison group (*n* = 49, 23.4%) at 3 months (RR = 2.10, *p* < 0.001); and 12 months follow-up (intervention: *n* = 57, 47.9%; comparison: *n* = 60, 30.8%; RR = 1.52, *p* = 0.006). Remission (PHQ-9 ≤ 5) was greater in the intervention group (*n* = 32, 26.9%) than comparison group (*n* = 33, 16.9%) at 12 months (RR = 1.72, *p* = 0.016).

**Conclusion:**

A task shared collaborative stepped care model can improve detection of CMDs and reduce depressive symptoms among patients with chronic conditions under real world conditions.

## Background

Mental disorders are on the rise globally, and are often comorbid with other chronic conditions, being two to five times more prevalent in people with chronic physical health conditions than the rest of the population [1–4]. Mental-physical comorbidities are associated with greater decrements in health outcomes [1] and increased health care utilization costs [5]. The need for chronic disease management to include treatment for co-existing common mental disorders (CMDs) is thus increasingly viewed as a priority in the global challenge of care for multi-morbidity [6].

South Africa is one of a growing number of low- and middle-income countries (LMICs) experiencing a rising burden of multi-morbid chronic conditions [7]. This is a consequence of the transition of HIV to a chronic condition with the scale-up of antiretroviral treatment; as well as the intensifying non-communicable disease (NCD) burden. In response the South African Department of Health has pioneered an Integrated Clinical Services Management (ICSM) approach that strives to service the majority of patients in primary health care (PHC) at a single delivery point using integrated clinical chronic care guidelines [8]. Integration of mental health care is part of ICSM in South Africa, but has been shown to be inadequate [9]; with a treatment gap of 75% for CMDs in South Africa [10]. While there is evidence of the effectiveness of collaborative care models for the treatment of common mental disorders (CMDs) comorbid with chronic physical conditions from high-income countries [11], there is little evidence of the effectiveness of task-shared collaborative care models for physical and mental multi-morbidity from LMICs.

The aim of this study was to evaluate an integrated collaborative care package of care for chronic patients with co-existing depressive and alcohol use disorder (AUD) symptoms that strengthened identification and management of these CMDs under real world conditions through strengthened referral pathways in one case study district in South Africa [12]. The study forms part of the PRogramme for Improving Mental Health CarE (PRIME) research consortium concerned with the development, implementation and evaluation of integrated packages of care for priority mental disorders at PHC level in five LMIC countries [13]. The specific objectives of this study were to assess primary outcomes of whether the collaborative care package: i) improved provider identification of depressive and AUD symptoms in chronic care patients and ii) reduced depressive symptoms and improved functioning in screen positive chronic patients identified and referred for care within the task shared stepped up collaborative care model. Secondary outcomes assessed effects in relation to health equity criteria.

## Methods

### Setting

The study site was in the Matlosana municipality in Dr. Kenneth Kaunda District (DKK) in the North West Province. The district population was estimated to be approximately 796,823 at the time of the study, while the catchment areas where this study was located comprised over 90,000 people serviced by four primary health care facilities varying in size, serving between 2353 and 6058 chronic care patients per month. Further details of the DKK district can be found elsewhere [12].

### Description of the collaborative mental health care package

Details of the collaborative care package that was developed through the formative phase and evaluated by this study are described in greater detail in Petersen et al. [12]. In brief, it comprised the following five components: i) PHC nurses functioned as case managers and were oriented to the ICSM, trained in clinical communication skills to facilitate person-centered care, and provided with supplementary mental health training in basic adult care guidelines (known as Adult Primary Care in South Africa) [14]; ii) Doctors were oriented to the importance of mental health and upskilled to prescribe antidepressant medications; iii) Referral pathways for psychosocial counselling for patients with mild to moderate depressive symptoms were strengthened with the introduction of clinic-based lay counsellors trained and supervised to deliver individual and group-based counselling drawing on cognitive behavioural therapy techniques which have international evidence of effectiveness [15]; and v) A referral form to monitor nurse referrals to the counsellor was introduced.

### Research design

The research design was pragmatic with the intervention delivered independently of the evaluation. Given the complex nature of the collaborative care package, the evaluation comprised two main components: i) a Facility Survey to assess effects of the intervention on provider detection of depression and AUD; and ii) a non-randomly assigned comparison group cohort study to assess changes in symptom severity and functioning among screen positive patients identified and referred for further care by the PHC nurses compared to those not identified.

### Facility detection survey (FDS)

#### Study procedure, sample and measures

The primary objective of the Facility Detection Study was to estimate the change in detection of depression and of AUD by clinicians serving the adult chronic care population in intervention clinics. The design and power calculations for the sample sizes are described in greater detail elsewhere [16]. The FDS was conducted in three of the four facilities where the mental health APC module had not yet been implemented. The study procedures used at baseline and follow-up FDS were the same, with independent samples recruited in each study round. The baseline FDS was conducted from February–April 2014, before the implementation of the intervention began in April 2014. Training and embedding continued to September 2014. The follow-up FDS was 12 months after completion of the embedding period (October–December 2015) (see Fig. 1).

Adult patients were recruited from the chronic care waiting areas of the three PHC facilities by trained recruiters who gave short oral presentations on the study. No reference to mental health was made to minimize sampling bias and limit stigma. Eligibility of patients who volunteered was confirmed in a private space and study objectives discussed. Eligibility criteria were: 18 years or older; attending the clinic for treatment for a chronic illness (e.g., HIV, tuberculosis, hypertension, etc) and capacity to understand the questions posed in either seTswana (the dominant local language) or English. Written informed consent was obtained from literate participants and illiterate participants consented by marking the form with a cross; with a witness countersigning.

Trained fieldworkers administered a structured questionnaire, programmed into mobile devices. Questions included items on demographic characteristics, treated chronic condition(s), and screening instruments for probable AUD and depression. The Alcohol Use Disorder Identification Test (AUDIT), validated for use in South Africa [17], was used to screen for probable AUD, with participants scoring ≥16 considered positive for probable AUD, given nurse guidelines to provide advice for harmful drinking and refer patients with dependent drinking. Cronbach’s alphas were 0.78 (baseline) and 0.74 (follow-up). For probable depression, we used the Patient Health Questionnaire-9 (PHQ-9), with a cut-off of ≥10 being previously validated on a primary care population in South Africa [18]. Cronbach’s alphas were 0.88 (baseline) and 0.86 (follow up).

All screen positive participants on either screening tool, as well as 15% of randomly selected screen-negative participants (both AUDIT and PHQ-9) were asked to return for an exit interview immediately following their clinical consultation.

Participants’ exit interview data were used to assess clinical detection on the day of the interview. Broad criteria were used to classify participants as detected for AUD given our experience of nurse reticence to make a diagnosis of AUD (patients who reported a diagnosis of harmful or dependent drinking, and/or a referral to specialist alcohol services, and/or who received advice about managing problems with drinking alcohol). Two classifications (narrow and broad) were used for detection of depression: narrow - a diagnosis of depression was reported; broad – diagnosis of depression and/or referral for psychosocial counselling reported. The latter was included given our experience that nurses did not always inform patients of a diagnosis of depression when referring patients. Anti-depressant medication prescribed was also assessed. Participants who reported being on current treatment for either condition were excluded from the analysis.

### Statistical analyses

The socio-demographic and clinical characteristics of the participants recruited over the two study rounds were analyzed using means and standard deviations for continuous measures and counts and proportions for categorical measures. For each study round we report the number of participants who screened positive on AUDIT and PHQ-9, the number of screen positive participants who completed the exit interview, and the proportion who were classified as having been detected for AUD (among AUDIT positive), or for depression (among PHQ-9 positive) using both narrow and broad criteria. For the equity analysis, where sufficient data were available, we assessed whether change in detection over time was equitable by sex and by household food security. Both these demographic variables were shown as having the highest odds ratios for depression/AUD comorbidity in the same population in a previous study [19]. For the inequity analysis, we used binomial regression models to estimate change in detection, and included the relevant interaction term into the models, with an interaction term *p*-value< 0.20 suggestive of inequity. Second, for the screen negative PHQ-9 participants who were randomly selected to complete the exit interview, we tabulated the number of depression diagnoses and anti-depressant medication prescription. When it was not possible to estimate the change in detection because of zero counts in the baseline round, we reported the proportion detected and 95% confidence interval for the follow up round only. Procedures to estimate proportions, 95% confidence intervals and *p*-values incorporated weights to adjust for the imbalance in clinic-level sample sizes between rounds [20].

### Depression cohort study

#### Study procedure, sample and measures

Details of the sample size calculation, recruitment, questionnaire design, data collection procedures, and analysis plan for the PRIME cohort studies have been described in detail by Baron et al. [21] A cohort study for AUD patients was not conducted in South Africa because of the very low levels of AUD identification by the providers in the baseline round of the FDS.

Eligible participants were at least 18 years old; receiving care for a chronic physical condition; screened positive for probable depression and/or had been identified and referred for depression care by the providers; had the capacity to provide informed consent and comprehend the interview; and did not have a diagnosis of AUD or psychosis (see Fig. 2).

Chronic care patients were informed about the study in the waiting areas of the clinics prior to their clinic consultations and informed written consent was obtained from volunteers. The same informed consent procedure was followed for low-literacy patients as described for the FDS. Immediately after their clinical consultations, individuals who had given their consent were screened using the PHQ-9, and asked whether, during the consultation, they had been identified as having depression and/or had been referred for care to a provider within the collaborative care model. Individuals who provided affirmative responses to these questions were enrolled into the depression treatment group, regardless of their PHQ-9 score. Individuals who did not provide affirmative responses to these post consultation questions, but who scored 10 or more on the PHQ-9 were enrolled into a comparison group. Comparison group participants who later received a depression diagnosis were re-enrolled in the treatment group, and only the treatment group data of these participants were analyzed (see Fig. 2).

Cohort study participants completed three assessments: at enrolment (baseline); 3 months and 12-months post-enrolment. Cohort recruitment and enrolment occurred from August 2014 to July 2015, and follow-up was conducted from November 2014 to September 2016 (see Fig. 1).

Mobile devices were used by trained seTswane/English speaking fieldworkers to administer the questionnaire in private spaces at the clinic or participants’ homes. Each assessment comprised a range of demographic, clinical, health care use, social, economic, food security, and stigma-related measures. These are described in greater detail by Baron et al. [21]. Only measures pertaining to the socio-demographic and clinical characteristics of the sample are reported here.

Primary outcomes of the cohort study were response on the PHQ-9, defined as at least 50% reduction in score from baseline to 3 months and 12 months follow-up; and remission on the PHQ-9 at both follow-up visits, defined as a score of 5 or less - used as measures of clinically significant improvement in treatment trials using the PHQ-9, e.g. Huijbregts et al. (2013) [22]. Functional impairment, was assessed at the three time points using the 12-item WHO Disability Assessment Schedule (WHODAS 2.0) - previously used in South Africa amongst older and HIV populations [23]. Item response theory (IRT)-based scoring was used, with scores ranging from 0 to 100, with a higher score indicating greater functional impairment.

#### Analysis

Given the non-normal distribution of the sample’s demographic and clinical characteristics, baseline characteristics of participants in the treatment and comparison groups were compared using non-parametric tests – the Mann-Whitney U test for continuous measures, and Exact Fisher’s test for categorical variables. The mean symptom severity and functioning were compared between groups at each follow-visit. Given that neither measure was normally distributed at follow-ups, a multilevel mixed effect negative binomial regression was used, controlling for HIV status and recruitment clinic, as these were imbalanced at baseline [21]. Risk ratios for the primary outcomes (i.e. 50% reduction in scores at 3 months and 12 months follow-up, as well as remission) were assessed using a modified Poisson regression, with robust variance estimator, as binomial models failed to converge [24]. Again, the models were adjusted for demographic or other clinical differences between the comparison and treatment groups at baseline. To assess equity in primary outcomes across gender and household food security, the same negative binomial regressions were conducted, this time including either gender or household food security at baseline as an interaction term.

## Results

### Facility detection survey

In the first round, 1322 participants were eligible and consented to participate in the study. Twelve of these were on current treatment for depression and excluded from the study, resulting in a total of 1310 participants at baseline. During the second round, 1257 were eligible and consented to participate in the study; of these 11 were found to be on current treatment for depression and excluded, resulting in a total of 1246 participants at follow-up. The demographic and clinical characteristics of the Facility Detection Survey participants are presented in Table 1.

**Table 1:** Demographic and clinical characteristics of participants in PRIME implementation clinics, Matlosana sub-district, Northwest Province, South Africa, 2014 & 2015

	Pre-implementation (*n* = 1310)	Post-implementation (*n* = 1246)	p
Age, years (SD)	46.9 (13.3)	46.2 (13.5)	0.18
Female (%)	983 (75.0)	967 (77.6)	0.14
Marital status (%)			0.20
Single	405 (30.9)	358 (28.7)	
Partnered	619 (47.2)	581 (46.6)	
Divorced/Separated/Widowed	286 (21.8)	307 (24.6)	
Education (%)			0.07
Never attended school	89 (6.8)	76 (6.1)	
Some primary	320 (24.4)	261 (20.9)	
Completed primary	720 (55.0)	705 (56.6)	
Completed secondary	181 (13.8)	204 (16.4)	
Employment Status (%)			0.01
Unemployed	519 (39.6)	513 (41.2)	
Paid	505 (38.5)	521 (41.8)	
Other^a^	286 (21.8)	212 (17.0)	
Household food insecurity (%)	272 (20.8)	538 (43.2)	0.01
Clinic (%)			0.01
Grace Mokhomo	583 (44.5)	495 (39.7)	
Kanana	274 (20.9)	342 (27.4)	
Orkney	453 (34.6)	409 (32.8)	
Chronic condition (%)			
HIV	783 (59.8)	781 (62.8)	0.12
Hypertension	671 (51.2)	664 (53.4)	0.28
Diabetes	123 (9.4)	113 (9.1)	0.84
Arthritis	53 (4.1)	44 (3.5)	0.54
Chronic respiratory disease	56 (4.3)	31 (2.5)	0.02
Tuberculosis	73 (5.6)	13 (1.1)	0.01
Epilepsy	47 (3.6)	29 (2.3)	0.06
Screening (%)			
PHQ-9 Screen Positive	107 (8.1)	130 (10.3)	0.056
AUDIT Screen Positive	43 (3.2)	63 (5.0)	0.03

P-value calculated with Fisher’s exact

^a^Voluntary, student, retired/pensioner, other

Across both survey rounds, the mean age of the sample was 46 years; approximately three-quarters were women; the majority were receiving care for HIV or hypertension. There were significant between-round differences in participants’ employment status, food security, clinic site, and AUDIT screening.

For depression, as seen in Table 2(a), the pre-implementation diagnosis of depressive symptoms was 5.2% using the narrow definition, and 14.2% using the broader definition. Post-implementation, using the narrow definition, detection of depressive symptoms reached 16.2%, an increase of 11.0% (95% CI 3.3, 18.6); using the broad definition, detection increased to 26.7%, an increase of 12.5% (95% CI 1.9, 23.0). In the inequity analysis, the change in detection for food secure participants, using the broad definition, increased to 33.8%, compared to 23.0% for food insecure participants (*P* = 0.080). There was no evidence of inequity (*P* = 0.656) for men versus women participants.

**Table 2:** Detection of Depression and Alcohol Use Disorder among chronic care patients in PRIME implementation clinics, Matlosana sub-district, Northwest Province, South Africa, 2014 & 2015

Disorder	Pre-implementation	Post-implementation	Detection difference (95% CI)^a^
a			
Depression			
Screened	1310	1246	
Screen positive	106/1310	124/1246	
Detection data available ^c^	102/106	116/124	
Detection-narrow*	6/102 (5.2)	19/116 (16.2)	+ 11.0 (3.3, 18.6)^a^
Detection-broad**	14/102 (14.2)	31/116 (26.7)	+ 12.5 (1.9, 23.0)^a^
Alcohol use disorder			
Screened	1310	1246	
Screen positive	42/1310	62/1246	
Detection data available ^c^	37/42	58/62	
Detection	0/37 (0.0)	8/58 (11.7)	11.7 (0.6, 22.8)^b^
b. Screen negative patients			
Screened	1310		1246
Probable non-case ^d^	1084/1310		996/1246
Randomly Selected	118/1084		113/996
Detection data available	110/118		109/113
Depression			
Detection-narrow ^e^	1/110 (0.9)		2/109 (1.9)
Detection-broad ^f^	8/110 (6.7)		8/109 (7.5)
Antidepressant prescribed	2/110 (1.6)		0/109 (0.0)
Alcohol use disorder			
Detection	2/110 (1.6)		0/109 (0.0)

Counts are reported as observed, while proportions, differences, 95% confidence intervals and *P*-values are estimated with clinic-based weights

^a^Estimated with binomial regression

^b^Estimated as one-sample proportion

^c^Completed post-consultation exit questionnaire

^d^Screen negative on PHQ-9 and AUDIT, and no depression symptoms over the past 12 months

^e^Reported diagnosis of depression

^f^Reported diagnosis or referral for psychosocial counselling

*Reported diagnosis of depression

**Reported diagnosis or referral for psychosocial counselling

For AUD, no AUDIT-positive participants were detected in the pre-implementation round. In the post-implementation round, 11.7% (95% CI 0.6, 22.8) were detected. There were insufficient data for conducting inequity analyses for AUD detection and treatment.

As seen in Table 2(b), of the 1310 participants enrolled in the pre-implementation round, 1084 screened negative on both PHQ-9 and AUDIT. Of the 1084, 118 were selected randomly to complete the exit interview, and 110 were successfully interviewed after their consultations. Of the 110, 0.9% had been detected and treatment initiated for depression using the narrow definition; and 6.7% using the broad definition. For AUD, 1.6% were prescribed anti-depressant medication and 1.6% were diagnosed with AUD. These proportions did not change significantly (all *P* > 0.05) between the pre- and post-implementation surveys.

### Depression cohort study

Of 2602 patients screened, a total of 453 participants were enrolled in the cohort study. An initial 205 patients were diagnosed with depression and recruited into the treatment group. Another 248 patients were not diagnosed but screened positive on the PHQ-9, and were recruited into the comparison group; of these, 12 participants were subsequently diagnosed with depression at a follow-up visit at the clinic, and re-enrolled into the treatment group. The final sample was 236 for the comparison group and 217 for the treatment group (see Fig. 2).

There were 82 participants (18.1%) lost to follow-up, mostly because of relocation and refusals. In the intervention group, 88.9% (*n* = 193) participants were followed at 3 months and 81.5% (*n* = 177) completed the 12-month assessment. In the comparison group, 88.6% (*n* = 209) completed the midline assessment and 82.6% (*n* = 195) the end-line assessment (see Fig. 1).

Of the 217 participants in the treatment group, 80 participants screened below the clinical cut-off of 10 on the PHQ-9 at baseline. These participants were excluded from the analysis, to ensure that both treatment and control groups were comparable. These participants did not differ from those included in the analyses in terms of demographic characteristics at recruitment, besides food insecurity (Table 3). The final sample included in the analysis comprised 373 participants: 137 and 236 in the treatment and comparison groups, respectively.

**Table 3:** Baseline demographic characteristics of the depression control and treatment cohorts included in the analysis

	Excluded from analysis ^a^	Included in the analysis		
	Treatment (*n* = 80)	Treatment (*n* = 137)	Comparison (*n* = 236)	*p*
Age, years (mean, SD)	44.8 (14.77)	42.6 (13.22)	44.0 (12.64)	0.469
Female (N, %)	61 (76.3%)	114 (83.2%)	190 (80.5%)	0.581
Marital status (N, %)				0.909
Single	22 (27.5%)	55 (40.2%)	90 (38.1)	
Partnered	46 (57.5%)	59 (43.1%)	103 (43.6%)	
Widowed/divorced	12 (15.0%)	23 (16.8%)	43 (18.2%)	
Education (N, %)				0.405
No or some primary	24 (30.0%)	38 (27.7%)	60 (25.4%)	
Completed primary	38 (47.5%)	80 (58.4%)	152 (64.4%)	
Completed secondary	18 (22.5%)	19 (13.9%)	24 (10.2%)	
Employment Status (%)				0.305
Unemployed	56 (70.0%)	102 (74.5%)	187 (78.2%)	
Paid	24 (30.0%)	35 (25.5%)	49 (20.8%)	
Food insecurity				
No	60 (75.0%)	62 (45.3%)	102 (43.2%)	0.746
Yes	20 (25.0%)	75 (54.7%)	134 (56.8%)	
Clinic (%)				0.005*
OR	10 (12.5%)	11 (8.0%)	21 (8.9%)	
KA	16 (20.0%)	32 (23.4%)	93 (39.4%)	
GM	38 (47.5%)	67 (48.9%)	77 (32.6%)	
MJ	16 (20.0%)	27 (19.7%)	45 (19.1%)	
Chronic condition (%)				
HIV	26 (32.9%)	57 (41.6%)	173 (73.3%)	< 0.001**
Hypertension	31 (39.2%)	53 (38.7%)	107 (45.3%)	0.233
Diabetes	3 (3.80%)	6 (4.4%)	15 (6.4%)	0.492
Other	9 (11.4%)	17 (12.4%)	24 (10.2%)	0.498
Main outcomes				
PHQ-9 (mean, SD)	6.3 (2.15)	14.5 (3.47)	12.8 (3.01)	< 0.001**
WHODAS (mean, SD)	24.1 (17.97)	37.6 (17.19)	40.0 (19.48)	0.251

^a^ Diagnosed but scored < 10 on PHQ-9

*p = < 0.01; **p = < 0.001

Participants in the comparison and treatment groups who were included in the analyses differed in terms of clinic of recruitment and in HIV status (Table 3). Also, participants recruited into the treatment group had significantly higher PHQ-9 scores (mean = 14.5, SD = 3.47), compared to participants in the comparison group (mean = 12.8, SD = 3.01).

Results of the modified Poisson regressions are presented in Table 4. The proportion of participants showing at least a 50% reduction in PHQ-9 scores from baseline to the 3-month follow-up was greater in the treatment group (*N* = 69, 55.2%) than in the comparison group (*N* = 49, 23.4%; RR = 2.10, *p* < 0.001). The rate of participants who showed remission on the PHQ-9 (score < 5) was also greater in the treatment group (*N* = 40, 32.0%) compared to the comparison group (*N* = 25, 12.0%; RR = 2.78, p < 0.001). The same significant trends were found at the 12-month follow-up, with 57 (47.9%) participants in the treatment group reporting at least 50% reduction in PHQ-9 score compared to baseline, and 32 (26.9%) participants scoring below 5, compared to 60 participants (30.8%; RR = 1.52, *p* = 0.006) and 33 participants (16.9%; RR = 1.72, *p* = 0.016) in the comparison group, respectively.

**Table 4:** PHQ-9 and WHODAS outcomes over time and across cohort groups

	Control group			Treatment group			adjusted *β* or *RR* ^*d*^ *(95%CI)*	*p*
	N	Mean score (SD) or N (%)	Adjusted mean change (95%CI) from baseline	N	Mean score (SD) or N (%)	Adjusted mean change (95%CI) from baseline		
Midline (3 months)^a^								
Response on PHQ-9 ^b^	209	49 (23.4%)	–	125	69 (55.2%)	–	2.10 (1.53 to 2.88)	< 0.001
Remission on PHQ-9 ^c^	209	25 (12.0%)	–	125	40 (32.0%)	–	2.78 (1.70 to 4.54)	< 0.001
PHQ-9 score	209	9.6 (4.66)	-2.63 (− 3.34 to − 1.92)	125	8.0 (6.16)	−5.05 (− 6.02 to − 4.08)	− 2.42 (− 3.49 to − 1.34)	< 0.001
WHODAS score	209	34.7 (21.14)	− 4.65 (−8.02 to − 1.28)	125	23.6 (20.99)	− 11.11 (− 15.18 to − 7.05)	− 6.46 (− 11.37 to − 1.55)	0.010
Endline (12 months)^a^								
Response on PHQ-9 ^b^	195	60 (30.8%)	–	119	57 (47.9%)	–	1.52 (1.13 to 2.05)	0.006
Remission on PHQ-9 ^c^	195	33 (16.9%)	–	119	32 (26.9%)	–	1.72 (1.11 to 2.68)	0.016
PHQ-9 score	195	9.0 (4.89)	−3.10 (− 3.83 to − 2.37)	119	7.9 (5.17)	−5.07 (− 6.05 to − 4.09)	−1.97 (− 3.04 to − 0.90)	< 0.001
WHODAS score	195	31.3 (22.39)	−7.20 (− 10.68 to − 3.73)	119	28.4 (21.64)	−8.00 (− 12.02 to − 3.99)	−0.80 (− 5.68 to 4.07)	0.746

^a^ Comparison group is the reference group; ^b^ defined as at least 50% reduction in score at follow-up compared to baseline; ^c^ defined as PHQ-9 < 5 at follow-up; ^d^ RR = Risk ratio

Change in mean scores on the PHQ-9 and WHODAS over time are also presented in Table 4. After controlling for HIV status and clinic of recruitment, the mixed effects analyses reveal a significant difference between the two groups at the 3-month follow-up; a greater decrease in PHQ-9 scores in the treatment group (M = -5.05, 95%CI: -6.02 to − 4.08) compared to the comparison group (M = -2.63, 95%CI: -3.34 to − 1.92; *β* = − 2.42, *p* < 0.001) and a greater decrease in WHODAS scores from baseline in the treatment group (M = -11.11, 95%CI: -15.18 to − 7.05) compared to the comparison group (M = -4.65, 95%CI: -8.02 to − 1.28; *β* = − 6.46, *p* = 0.010). A similar trend was seen for the PHQ-9 scores at the 12-month follow-up compared to baseline (treatment: M = -5.07, 95%CI: -6.05 to − 4.09; comparison: M = -3.10, 95%CI: -3.83 to − 2.37, *β* = − 1.97, *p* < 0.001). The change in WHODAS scores at the 12-month follow-up was not significantly different between the two groups.

Inequity analyses of the impact of the intervention on outcomes by gender and household food security are presented in Table 5. There is no evidence of inequity by gender at either follow-up time points. At 3 months follow-up, however, the intervention was found to have a significantly more positive effect on reducing depressive scores among participants who reported being food secure at baseline (*β =* − 3.73, 95%CI -5.30 to − 2.15) (M = − 6.62, 95% CI: -7.95—5.28) compared to those reporting being food insecure (*β = − 1.23, 95%CI -2.65 to 0.20; β = − 2.50, p < 0.05).* This trend persisted at 12 months follow-up.

**Table 5:** Equity analysis of Cohort Study

	Control group			Treatment group			Difference of difference	adjusted *β (95%CI)*
	N	Mean PHQ-9 score (SD)	Adjusted mean change in PHQ-9 (95%CI) from baseline	N	Mean PHQ-9 score (SD) or N (%)	Adjusted mean change in PHQ-9 (95%CI) from baseline		
Midline (3 months)								
Sex								
Male	43	8.3 (4.19)	−3.56 (− 5.04 to − 2.08)	21	6.8 (4.98)	−5.39 (− 7.45 to − 3.33)	− 1.83 (− 4.28 to 0.62)	0.76 (− 1.94 to 3.45)
Female	166	9.9 (4.73)	−2.41 (− 3.18 to − 1.64)	104	8.2 (6.36)	−5.00 (− 6.03 to − 3.97)	−2.59 (− 3.77 to − 1.41)	
Household food insecurity								
Food secure	89	8.7 (4.20)	−2.89 (− 3.92 to − 1.86)	58	6.2 (4.84)	− 6.62 (− 7.95 to − 5.28)	−3.73 (− 5.30 to − 2.15)	− 2.50 ^a^ (− 4.60 to − 0.41)
Food insecure	120	10.3 (4.88)	−2.56 (− 3.47 to − 1.65)	67	9.5 (6.79)	−3.79 (− 4.98 to − 2.59)	−1.23 (− 2.65 to 0.20)	
Endline (12 months)								
Sex								
Male	38	7.3 (4.29)	−4.44 (− 5.94 to − 2.94)	21	7.7 (4.34)	− 4.64 (− 6.72 to − 2.56)	−0.20 (− 2.67 to 2.28)	2.18 (− 0.55 to 4.91)
Female	157	9.4 (4.95)	−2.80 (− 3.59 to − 2.01)	98	8.0 (5.35)	−5.17 (− 6.22 to − 4.13)	−2.38 (− 3.55 to − 1.20)	
Household food insecurity								
Food secure	86	8.3 (4.81)	−3.27 (− 4.31 to − 2.24)	55	6.5 (4.97)	−6.42 (− 7.76 to − 5.08)	−3.15 (− 4.72 to − 1.58)	− 2.24 ^a^ (− 4.34 to − 0.14)
Food insecure	109	9.6 (4.91)	− 3.10 (− 4.04 to − 2.17)	64	9.2 (5.06)	−4.01 (− 5.22 to − 2.81)	−0.91 (− 2.33 to 0.52)	

^a^ Significant at < 0.05 level

## Discussion

The results of the FDS suggest an improvement in nurse-detection and treatment initiation of depressive and AUD symptoms following implementation of the integrated collaborative care package in the district. Notably, identification remained essentially absent for individuals who were probable non-cases. In other words, the positive predictive value of nurse identification and treatment initiation was high for both depression and AUD.

The results of the cohort study indicate that patients correctly identified and referred for further care for their depressive symptoms had a greater chance of having a clinically significant reduction in depressive symptoms to the point of being in remission at both 3 and 12 month follow-up than those not referred. They also showed a significant reduction in functional disability at 3-month follow-up compared to the non-intervention group, although this effect was not sustained at 12 months. The intervention was significantly more successful with food secure participants, suggesting that food insecurity, may serve as a barrier to improvement in depressive symptoms; being associated with poverty in more urban areas in South Africa [25]. This adds to the growing body of evidence suggesting the need for accompanying income generating initiatives for people with depressive symptoms from poor socio-economic contexts [26].

While an improvement in the correct identification and treatment initiation of patients with depressive and AUD symptoms was noted, the treatment gap post training still remained large, with 75% of probable cases of depression and 84% of probable cases of AUD still not detected at 12 months follow-up. Previous studies suggest that training alone may be insufficient to improve identification of CMDs in PHC [27]. Other factors contributing to this gap resonate with our understanding of possible reasons and include individual provider level factors - with psychiatric stigma as well as providers’ own personal unresolved problems previously shown to act as barriers to the identification of emotional problems in patients [28, 29]. The inclusion of anti-stigma interventions [28], stress management and debriefing sessions to assist PHC personnel to engage in emotional labour has been previously suggested to assist integration efforts [29]. Further, the need for change management processes to accompany organizational changes associated with integrated care has also previously been highlighted [8].

### Limitations

There are a number of limitations of this evaluation. With regard to the FDS: i) Non-random sampling compromised the representativeness of the sample. An imbalance in the demographics between the two rounds was noted – with an upward trend in food insecurity, unemployment and alcohol screen positives in the second round - partially explained by retrenchments on the mines between the two survey rounds, with mining being a major industry and source of employment in the study site; ii) There were no control clinics involved in the FDS – with the possibility that the some of the improvements in detection were due to other factors in the health system; iii) An upwards bias in detection may have been introduced given patients’ heightened awareness of their potential symptoms through exposure to the survey interview prior to their consultation - although offset by being the case for both rounds as well as having screen-negative cases; and iv) The FDS only assessed clinical detection on the day of the interview – with diagnosis of a CMD generally taking several visits [27] – thus potentially under-representing detection rates. In relation to the cohort study, recruitment into the intervention and control arm was not randomized, thus increasing potential for bias from unknown confounders, although partially mitigated by multi-variable analyses.

## Conclusion

This evaluation shows that the PRIME-SA intervention package assisted PHC teams to identify and manage comorbid CMDs in chronic patients under real world conditions using a collaborative stepped care model. There was an improvement in accurate detection of CMDs by PHC nurses, and a reduction in depressive symptoms in patients identified with comorbid depression and referred for care. Further research is required to assess patient outcomes in patients with comorbid AUD. In the face of the negative impact that comorbid CMDs have on treatment adherence and overall health outcomes in chronic patients, this collaborative task shared model provides a potential model for mental physical multi-morbid disease management in South Africa and other LMICs in the context of specialist resource shortages. Additional findings will be provided by two parallel pragmatic cluster randomized control trials that are currently underway, testing effectiveness of this model on depression and physical outcomes (viral load suppression in HIV patients and reductions in blood pressure in hypertensive patients) in chronic care patients [30, 31].

## Abbreviations

AUD: Alcohol Use Disorder
AUDIT: Alcohol Use Disorder
CMD: Common Mental Disorder/s
DKK: Dr. Kenneth Kaunda district
FDS: Facility Detection Survey
ICSM: Integrated Clinical Services Management
IRT: Item response theory
LMICs: Low -middle-income countries
NCD: Non communicable disorder
PHC: Primary Health Care
PRIME: PRogramme for Improving Mental health carE
WHODAS 2.0: World Health Organization Disability Assessment Schedule

## References

[1] Moussavi S, Chatterji S, Verdes E, Tandon A, Patel V, Ustun B (2007). Depression, chronic diseases, and decrements in health: results from the world health surveys. Lancet..

[2] Ciesla JA, Roberts JE (2001). Meta-analysis of the relationship between HIV infection and risk for depressive disorders. Am J Psychiatry.

[3] Neuman MG, Schneider M, Nanau RM, Parry C (2012). Alcohol consumption, progression of disease and other comorbidities, and responses to antiretroviral medication in people living with HIV. AIDS research and treatment.

[4] Lonnroth K, Williams BG, Stadlin S, Jaramillo E, Dye C (2008). Alcohol use as a risk factor for tuberculosis - a systematic review. BMC Public Health.

[5] Naylor C, Parsonage M, McDaid D, Knapp M, Fossey M, Galea A (2012). Long-term conditions and mental health. The cost of co-morbidities.

[6] Patel V, Belkin GS, Chockalingam A, Cooper J, Saxena S, Unutzer J (2013). Grand challenges: integrating mental health services into priority health care platforms. PLoS Med.

[7] Oni T, Youngblood E, Boulle A, McGrath N, Wilkinson RJ, Levitt NS (2015). Patterns of HIV, TB, and non-communicable disease multi-morbidity in peri-urban South Africa- a cross sectional study. BMC Infect Dis.

[8] Mahomed OH, Asmall S (2015). Development and implementation of an integrated chronic disease model in South Africa: lessons in the management of change through improving the quality of clinical practice. Int J Integr Care.

[9] Fairall LR, Folb N, Timmerman V, Lombard C, Steyn K, Bachmann MO (2016). Educational outreach with an integrated clinical tool for nurse-led non-communicable chronic disease Management in Primary Care in South Africa: a pragmatic cluster randomised controlled trial. PLoS Med.

[10] Seedat S, Stein DJ, Herman A, Kessler R, Sonnega J, Heeringa S (2008). Twelve-month treatment of psychiatric disorders in the south African stress and health study (world mental health survey initiative). Soc Psychiatry Psychiatr Epidemiol.

[11] Archer J, Bower P, Gilbody S, Lovell K, Richards D, Gask L (2012). Collaborative care for depression and anxiety problems. Cochrane Database Syst Rev.

[12] Petersen I, Fairall L, Bhana A, Kathree T, Selohilwe O, Brooke-Sumner C (2016). Integrating mental health into chronic care in South Africa: the development of a district mental healthcare plan. Br J Psychiatry.

[13] Lund C, Tomlinson M, De Silva M, Fekadu A, Shidhaye R, Jordans M (2012). PRIME: a programme to reduce the treatment gap for mental disorders in five low- and middle-income countries. PLoS Med.

[14] Fairall L, Bateman E, Cornick R, Faris G, Timmerman V, Folb N (2015). Innovating to improve primary care in less developed countries: towards a global model. BMJ Innov.

[15] Singla DR, Kohrt BA, Murray LK, Anand A, Chorpita BF, Patel V (2017). Psychological treatments for the world: lessons from low- and middle-income countries. Annu Rev Clin Psychol.

[16] De Silva MJ, Rathod SD, Hanlon C, Breuer E, Chisholm D, Fekadu A (2016). Evaluation of district mental healthcare plans: the PRIME consortium methodology. Br J Psychiatry.

[17] Myer L, Smit J, Roux LL, Parker S, Stein DJ, Seedat S (2008). Common mental disorders among HIV-infected individuals in South Africa: prevalence, predictors, and validation of brief psychiatric rating scales. AIDS Patient Care STDs.

[18] Cholera R, Gaynes BN, Pence BW, Bassett J, Qangule N, Macphail C (2014). Validity of the patient health Questionnaire-9 to screen for depression in a high-HIV burden primary healthcare clinic in Johannesburg, South Africa. J Affect Disord.

[19] Petersen I, Rathod S, Kathree T, Selohilwe O, Bhana A. Risk correlates for physical-mental multimorbidities in South Africa: a cross-sectional study. Epidemiology and psychiatric sciences. 2017. p. 1–9. 10.1017/S2045796017000737. 10.1017/S2045796017000737 PMC6998962 29198237

[20] Hennekens CH, Buring JF (1987). Epidemiology in medicine.

[21] Baron EC, Rathod SD, Hanlon C, Prince M, Fedaku A, Kigozi F (2018). Impact of district mental health care plans on symptom severity and functioning of patients with priority mental health conditions: the Programme for improving mental health care (PRIME) cohort protocol. BMC Psychiatry.

[22] Huijbregts KM, de Jong FJ, van Marwijk HW, Beekman AT, Ader HJ, Hakkaart-van Roijen L (2013). A target-driven collaborative care model for major depressive disorder is effective in primary care in the Netherlands. A randomized clinical trial from the depression initiative. J Affect Disord.

[23] Hanass-Hancock J, Myezwa H, Carpenter B (2015). Disability and living with HIV: baseline from a cohort of people on long term ART in South Africa. PLoS One.

[24] Zou G (2004). A modified poisson regression approach to prospective studies with binary data. Am J Epidemiol.

[25] Grobler WCJ (2016). Perceptions of poverty: a study of food secure and food insecure households in an urban area in South Africa. Procedia Economics and Finance.

[26] Rao K, Vanguri P, Premchander S (2011). Community-based mental health intervention for underprivileged women in rural India: an experiential report. Int J Family Med.

[27] Thielke S, Vannoy S, Unutzer J (2007). Integrating mental health and primary care. Primary care.

[28] Egbe CO, Brooke-Sumner C, Kathree T, Selohilwe O, Thornicroft G, Petersen I (2014). Psychiatric stigma and discrimination in South Africa: perspectives from key stakeholders. BMC Psychiatry..

[29] Petersen I (2000). Comprehensive integrated primary mental health care for South Africa. Pipedream or possibility?. Soc Sci Med.

[30] Fairall L, Petersen I, Zani B, Folb N, Georgeu-Pepper D, Selohilwe O (2018). Collaborative care for the detection and management of depression among adults receiving antiretroviral therapy in South Africa: study protocol for the CobALT randomised controlled trial. Trials.

[31] Petersen I, Bhana A, Folb N, Thornicroft G, Zani B, Selohilwe O (2018). Collaborative care for the detection and management of depression among adults with hypertension in South Africa: study protocol for the PRIME-SA randomised controlled trial. Trials.

